# On the Increase of Insanity

**Published:** 1857-07-01

**Authors:** John Hawkes

**Affiliations:** Assistant Medical Officer, Wilts County Asylum


### Art. V.—ON THE INCREASE OF INSANITY.

BY JOHN HAWKES, M.R.C.S.,

Assistant Medical Officer, Wilts County Asylum.

The subject under consideration is both of national and social importance; it is one, too, that, with its increasing dimensions, claims the attention of all. The fair face of England, dotted over with her many public asylums for the relief and refuge of mental disease, presents a picture of rare and painful interest to the psychologist, philanthropist, and historian. If to such an accumulation of suffering be due, as some assert, the *apparent* increase of insanity, we may regard, not indeed lightly, but yet without particular anxiety, the existence of this mass of disease; but, on the other hand, if it be true, as we have reason to fear, that insanity, so far from being diminished, is actually on the increase, it then no longer becomes us to assume the attitude of indifferent spectators when mischief and ruin are brooding around our hearths and beside our social altars: rather should we resolve, as rational beings, to consider by what means the present unnatural mental phenomena may best be mitigated, and to discover if by any way the dark cloud that threatens posterity may happily be turned aside. The symptoms of disordered intellect, known under the general phrase of insanity, have been familiar to the world for ages; assuming at various times distinctive features, borrowed from the prevailing spirit of the epoch; encouraged at different periods by some peculiar existing superstitions; or carried as an epidemic disease, by reason of its contagious properties, from one country to another; affording proofs of the susceptibility of the human mind to outward impressions, and painfully illustrating the weakness of our nature, with its proclivity to disease and decay. But if, from the early traditions of the world and the history of the middle ages, we learn that disorders of the mind, no less than diseases of the body, have from time immemorial dwelt among us, it may cause neither surprise nor pain to reflect that mental disease, in many aggravated forms, is still rampant in the land; yet I doubt if ever the history of the world, or the experience of past ages, could show a larger amount of insanity than that of the present day. It seems, indeed, as if the world was moving at an advanced rate of speed proportionate to its approaching end; as though, in this rapid race of time, increasing with each revolving century, a higher pressure is engendered on the minds of men and with this; there appears a tendency among all classes constantly to demand higher standards of intellectual attainment, a faster speed of intellectual travelling, greater fancies, greater forces, larger means, than are commensurate with reason and health.

And is it possible that the unnatural forcing system necessary to meet the demand can be otherwise than wholesome? Is it that, as all classes are so rapidly rising in the intellectual scale, knowledge is so generalized that individual powers are in abeyance? or is it that those springs of intellect are dry from whence arose a Bacon, a Shakspeare, or a Newton? Truly, "there were giants in those days." And yet we shall do well to consider if things are thus, what will the end be? What, in short, must we expect if a progressive deterioration of mental and bodily powers be symptomatic of the age; of succeeding ages; perhaps of all time? But let us trust this is not the case, though it is apparent that some physical alteration has crept over the *vis vitæ* of our own generation within the present century—a change, it may be sufficient to say, attacking the stamina of life, inducing a disposition to adynamia, and rendering necessary a departure from the former plan of treating febrile disorders. Now, it has been observed by those to whose hands are intrusted the treatment of mental disease, that these cases require the same precautions.

The raving maniac must be abundantly supplied with nutritious aliment; and as the blood recovers the elements of health, the physical frame, and its occupant, the mind, give good evidence of the efficacy of the treatment pursued. It is not my intention to follow this argument further than to point out a palpable source of many obscure phenomena occurring both in physical and psychical disease, induced by an unhealthy condition of the primary elements of life; and as bodily disorders which partake of this character are wont to leave their impress on the frame, so may we expect the same results from such as are purely mental. When this is proved to be the case, the neutral ground between the two is shortened, and we come to view them not as paradoxical, or, so to speak, polarized phenomena, but rather as intimately related the one to the other, springing up and bound together by the same unhappy laws, too often mutually dependent, near neighbours, or dwelling, alas! in the same mansion. It is a matter of no slight difficulty to determine, in all cases, the primary or predisposing cause of mental disorders, apart from an hereditary taint existing in the constitution, and where there is no positive mental deficiency from birth; for it is only too probable that, from a much earlier period than the actual manifestation of disease, the fuel has been laid; and it therefore becomes a matter of grave consideration for those who may have the power vested in their hands, not so much for the cure, but, which is of far greater import, the prevention of disease, that many carry about with them unsuspected, perhaps through a long lifetime, the dormant seeds of insanity. Waiving now the question of hereditary predisposition and congenital weakness, as before stated, there is yet a fearful balance of minds wholly free from either of the above infirmities, and yet doomed to break down and wear out prematurely in the terrible struggle of the age. Among the various classes which compose a community, there may appear at first some discrepancy in regard to the proclivity exhibited by each particular segment of society to diseases of the mind.

It might possibly be supposed that those whose means are sufficient to procure every earthly enjoyment—to whom, in short, the word want is unknown—are not likely to suffer in the same degree as others in a humble sphere; and yet in this particular class we find the strongest proof that hereditary honours and hereditary diseases descend together. But in seeking for other causes—as failure of bodily health, pecuniary embarrassments, over-anxiety, too great application to business, &c.—we must embrace a wider field of observation, such as is presented in the middle and lower walks of life: among those, for instance, who earn their bread by the sweat of *their brain;* whose lives are a continual struggle, whose daily toils are unmitigated by pleasure, to whom each morrow brings fresh cares, and night scarce brings repose. The hard-worked professional man, who spends his days in painful efforts to make two ends meet; the avaricious man of commerce, only aiming to double his gains and grind fresh profits from his wares; the speculating capitalist, eager to lay out his treasure in the best market; the adventurous merchant, whose temple is the counting-house. What a restless sea! What troubled waves of thought and care rise up even within this single catalogue of callings! But look we further. Observe the young of all classes, with what suicidal frenzy they commit themselves to sorrow. Whether, in obedience to the promptings of high ambition, weaving an entanglement of thought, and straining the sinews of the mind in attempting to achieve the gain of riper years, and to wrest the victory ere the battle has begun; or else, led spell-bound by passion, the powers and resources of youth are squandered in the bed of the voluptuary; sin sinking into the heart with all its accursed stains, polluting the fountains of reason at their source, and embittering the springs of life. Do we not here find predisposing causes with a vengeance, borne it may be long, but only waiting for the spark to fall? Leaving, for the present, the consideration of insanity as affecting the upper and middle classes, let us turn our attention to that multitudinous body which, ever accumulating out of the necessary changes and chances of time and circumstances, lies in a posture of decrepitude and state of slow decay at the base of the structure on which society is reared. Under the humble appellation of "paupers" will be found persons, whose phases of existence have been spent, it is probable, in widely different spheres. Some, nurtured in the lap of luxury, have learned in their old and helpless age to taste the cup of sorrow, exchanging scenes of the broadest splendour for the straight and narrow gate of poverty. The aged and respected couple who, after years of honest toil, when the day for work has passed, are compelled to eat the parish loaf in thankful resignation; the dissipated mechanic, once perhaps well to do, no longer able to earn a subsistence, after passing through various shades of misery, is reduced to swell their ranks. The broken-hearted widow; the young and helpless; the despairing, the destitute, live under this name. It is, in fact, a class composed of the tag-end and *débris* of society at large—a formation that has settled down out of the troubled waters of life into the stagnant obscurity of a workhouse. In this division of the community, causes most frequent among the upper classes are scarcely to be found. Ambition, pride, self-aggrandisement, intellectual labour, political excitement, &c., are almost wholly wanting: but we have instead intemperance very frequent; distress, in most cases arising from poverty; want, insufficient food, loss of work, anxiety as to maintenance, &c.—such are some of the most common causes of insanity in the poor. There is another, and by no means a rare cause, especially in certain districts, and which I shall have occasion to allude to further on—religious distress, or excitement. It is not always so easy as it seems to distinguish between the predisposing cause and that which is the immediate precursor of the disorder, and hence named the exciting. A man, for instance, is addicted to habits of intemperance, in which he has indulged, it may be, for several years; he is often intoxicated, more frequently, perhaps, than otherwise. At last some accident shall befall him, by which the already over-burdened nervous system receives a severe contusion; we will suppose a blow on the skull as the most common kind of injury; and after this the malady, long since impending, appears. Or exactly the same train of circumstances may be reversed: in this case from the blow, and consequent contusion inflicted on a healthy brain, disease is lighted up, at first probably insidious, perhaps only a slight deviation from accustomed routine, an alteration in social habits, or an eccentricity of manner never before noticed. Thus, we sometimes find a sober right-thinking man become addicted to drinking, or to frequent places of low resort; to adopt, in short, habits which before the accident he would have entirely repudiated; but the disease does not stop here: it is at present only incipient, but an early occasion of excess will at once draw aside the veil, and insanity in its worse form shall stand revealed. Another instance of the relation between predisposing and exciting causes may be given. A wife, of a soft, gentle, and affectionate disposition, has long been subjected to ill-treatment by her brutal husband; she has suffered long and endured patiently all his cruelty; at length a beloved child is taken away by death, and the mother's mind suddenly gives way; she falls into a state of melancholia, and will probably attempt self-destruction. Such cases are not, I fear, uncommon. When dwelling briefly on the predisposition to insanity often induced by reckless or vicious habits in the more intelligent classes, it was not judged necessary to make more than a single remark on the tendency thus encouraged.

Exciting causes are always present, as surely as the flash of electric light precedes the thunder of heaven; and, if duly investigated, the predisposing cause will generally be found. Among persons of this description, whose *morale* and *physique* are alike too often below par, trifling, or even ludicrous events, will suffice for an exciting cause. Thus, a man of low tastes, which he abundantly gratifies, to the cost both of physical and pecuniary resources, finds himself, to use a common phrase, under water, and he exists—for it does not deserve the name of life—in painful and degrading poverty: so far the train of mental disease has been carefully laid. It shall happen, by an unlooked-for accident, he suddenly becomes possessed of considerable property; the means for indulging every caprice and propensity are at once in his hand; but he straightwise goes mad: it was the exciting cause. A gentleman was some time ago under my care, suffering from chronic mania, induced by taking part in a political struggle in his native town. Here there was, indeed, a strong hereditary taint; but the fatal mistake which he made in sharing the excitement of the strife, proved the immediate cause of his malady. Even when no hereditary proclivity exists, there can be no doubt a predisposition may be formed by too great a strain on the mental forces sustained for an undue period. That pure and subtle element, the mind, unlike to visible matter, endures sharp trial and weighty stress of toil full long, without betraying to common eyes the greatness of its pain and sorrow; but there must come at last an hour when nature fails, and then, alas! too late, the secret is. revealed. It was thus with the gifted Chatterton; and many a child of genius, whose sun has gone down at noon, may be cited to prove the dire results of an overwrought mind. We know not—may we never learn—by what secret pangs the mind has been urged, day by day, to its fatal consummation. The memory of Kirke White will ever borrow from his mental state a shade of sadness; and in the present day this terrible truth has been lately enunciated by the untimely death of one great light of the age, the lamented Hugh Miller. It is no uncommon thing to find the most exquisitely-finished workmanship of nature's hand, alas! most prone to decay; to find a highly sensitive nervous system coincident with an active and powerful mind: the first painfully alive to every shock of the outer world, its every chord vibrating to those rude blasts that sweep, like the winds of Boreas, over the strings of an Æolian lyre; while the latter, supreme, exacting from a fragile and delicately-organized brain labours beyond its strength. Can we wonder at the consequences that ensue?

In some rare cases, where, like a strong man on a strong steed, the mind and brain are happily matched, we see with feelings of surprise and admiration how nobly the work is done, how well the weight is borne; for through many a year it carries on over ground where others before have fallen; still it travels on. Yet the good steed tires at last; his head droops, his flank heaves, he would fain be at rest; but the rider cares not for rest—the race is not yet won, and with impatient spur he urges onward; alas! the steed is spent, it can go no further, and sinks on the track. Have we not an example of this in the latter end of Southey and Walter Scott? But a highly sensitive nervous system is not necessarily conjoined with great mental power, for we find this even in persons of very ordinary intellectual capacity; and in such cases, when the mind is deficient in that general amount of tone and vigour proper to natural health, there is, if anything, still greater reason to apprehend the decay and loss of reason. We have not here a master-hand at the helm, but the vessel rides rudderless at the mercy of the waves: naturally weak, unstable, and infirm—little calculated to war with the elements, or to join conflict in the battle of life—the poor helpless craft soon becomes a wreck; the first heavy storm shakes her to the keel, and soon the shattered hull is tossed upon the shore.

It is sometimes difficult to discriminate between the cause and effect of mental maladies; thus, it not unfrequently happens that persons, from whose psychical idiosyncrasy particular caution should be observed in their daily habits of life, are unhappily prone to take the very road to destruction. One, who has perhaps in early life exhibited a disposition to convulsive affections, as he grows up, in spite of warnings, entreaties, and example, will obstinately persist in a career which must ultimately destroy him. It would seem, indeed, as if such unhappy individuals, though at the time in apparent enjoyment of their reason and right understanding, yet lack the self-governing faculty necessary to keep them from harm. In the case supposed, when a delicate infancy is safely passed and childhood ripening into youth—when health seems firm and danger far removed—then, as though in obedience to an uncontrollable impulse, by a spell which cannot be unbound, habits are taken up and pursuits greedily followed—sensual gratification becoming the sole object of life—till *apparently* from a course, either longer or shorter, of sustained profligacy, the mind breaks down.

In this case, the cause and effect may be easily misplaced; it is an example of the difficulties attending a correct elucidation of mental disease; indeed they can hardly be overrated. So insidiously planted are the seeds of insanity, that the manner and time of sowing it is often impossible to tell. A mother may rear a healthy family of five or six children, and the seventh shall prove an idiot from birth, or quickly lapses into a state of helpless fatuity.

Both parents may be healthy, and their ancestry, so far as is known, for too little is generally known in these matters by all parties whom it concerns. Well, the child is a mental cripple, and so he remains through life; but possibly, if the secret was discovered, it would show no fault with the forefathers of this *infelix proles;* it may have simply resulted from a transient abnormal, or at least unhealthy, condition of the mind in one or other parent, most probably the husband, nine months before birth. This affection, resulting perchance from inordinate anxiety, or intemperance, or some other weakness, has long since passed off and been forgot, but the print has been laid, the die cast, the seed sown.

On looking over most registers of causes, it will be observed that the exciting causes are more frequently chronicled than those that are simply predisposing; it is only natural that the *post hoc, ergo propter hoc*, argument should be followed in these cases; and, indeed, this is almost always the rule: such as are predisposing being often of the same kind, yet failing to produce a manifest effect are liable to pass unnoticed. In some instances no cause can be assigned; probably this often arises from ignorance and want of observation.

Anxiety occurs both as a predisposing and exciting cause; in most cases it is the latter, frequently supervening on the death of a husband, the sole support of the family, and generally arising from the difficulties that spring up to share with grief the bosom of the bereaved. Occasionally it is a predisposing cause, as anxiety to obtain a livelihood, coupled with the natural dread of poverty, a fear which, like the basilisk of old, draws its trembling victim to his doom. Poverty and want! What else could be expected? What more to the point for filling a pauper asylum?

The tendency of dissolute habits towards inducing aberration of the mind has been already mentioned; if prolonged habits of dissipation frequently predispose toward insanity, fits of intemperance prove not seldom the immediate exciting cause. There is little occasion for wonder that it should be so, when we take into consideration the peculiar influence exercised by alcohol on the general nervous system. The property which this substance possesses of inducing that delirious feeling of enjoyment that precedes absolute intoxication, together with its acknowledged virtues in restoring the failing force of life, in reviving, that is to say, the dying, and assuredly in many cases keeping death itself at bay, has gained for it such an universal ascendancy over the widely-differing tastes and habits of mankind, that it is to him at one time a fostering nurse, at another a cheering friend, again, a powerful ally, and lastly, an oppressive and cruel enemy. It cannot be gainsaid that alcohol is a great boon; through its power of quickening nervous energy it fans, so to speak, the fading spark of life into a warm diffusive glow, and though this may prove, too often, but the expiring gleam, it affords valuable time, oh how precious! to the dying person. Its restorative virtues are daily exercised in the sick, expediting their recovery; its genial influence in wholesome moderation may cement the bands of friendship and gain a noble cause; but abused, serpent-like, it bites the heel of those who unwittingly tread thereon; not the destroying pestilence nor the invader's sword shall prove so devastating, so relentless a foe. From being a gift, hallowed by divine authority, it becomes a fearful instrument in the hands of the devil for the destruction of the human race; truly may the advocates of temperance exclaim, "if war has slain her thousands, drink has slain its tens of thousands;" no longer an emblem of peace and prosperity, it becomes the dread token of misery and crime. What more lamentable, more pitiable vice can there be, than that by which man's proudest gift, his reason, is dethroned from its high seat by a cruel and enslaving tyrant, whose end is accomplished when that reason is for ever cast out; and what words of horror and surprise can describe the height and depth of folly that invites this king of terrors to come and take possession? Intemperance is a fertile source of insanity; it is not like sorrow, or disappointment, or, in fact, any other cause which may eventually be lived down, but in the habitual drunkard burns an inward, consuming fire. When one has abandoned himself wholly to this vice, it is impossible to say he may ever recover; the more urgent symptoms of disease may indeed be subdued, and to casual observers he may seem a new man; but should he be removed from those wholesome restraints, which have of necessity been placed on his appetite, he is sure to relapse, and in the emphatic language of Scripture, "the last state of that man is worse than the first." Superstition does not appear to be so common a cause of insanity as might be supposed among the lower classes of society. The prevailing superstitions of the day—spirit-rapping, table-turning, mesmerism, and animal magnetism—exercise their baneful influence on more susceptible minds than belong to the labouring population. These are rocks in the fathomless sea of mysticism, on which many an empty head has split, and many a shallow mind been stranded; but happily in this country these causes are rare, though so frequently met with in the wards of American asylums; instances in which the precocity of intellect, the hardihood of ignorance, with the recklessness of presumption, are brought into juxtaposition without the healthy counterpoise of sound knowledge, humility, and piety. The English mind, speculative, somewhat sceptical, but eminently practical, has turned away from such puerile fancies and silly diversions as our Transatlantic brethren have taken up and still practise. We care not to tickle our brains with such unnatural, irrational pursuits.* Society has shown herself slow to learn mere physical hygiene, and how much more that which is mental. But is not this as needful as the other? The robust, uncultivated mind may smile at the props and precautions so necessary for the highly wrought intellects of the educated and brain-tasked men of to-day, just as a wilderness, swept by the winds of heaven and unsullied by man's breath, can afford to dispense with well planned sewers and skilful officers of health, though for the closely populated town these are indispensable. And as among the crowded abodes of man, all adjuvants to sickness should carefully be expunged, so among the labyrinths of intellectual pursuit must we as sedulously remove, or at least avoid, those dangerous and insidious provocatives of disease that are broad-cast through all ranks of the people. With these few comments on the perils of vain, unphilosophical, and superstitious inquiries, I now proceed to the consideration of religious excitement, or ecstasy, in its various kinds, as a fertile source of mental derangement and decay.

A morbid state of the feelings, characterized by their transient exaltation, arising often by force of imitation, sometimes, it may be believed, involuntarily and under the unhealthy influence of intense fervour of a spiritual kind. Such is "religious excitement," as it is commonly and vaguely termed. This description of phrensy has been met with in many ages, and under widely different forms of religion. Its symptoms are very varying and inconstant; the reaction which ensues may completely overshadow the preceding excitement and render its existence doubtful; in many cases it may be questioned if the mind is really affected till the second stage of depression has set in; in others, whether the extreme excitement may not be ascribed to actual and positive insanity, passing away with the cause, and leaving the individual sane, though more prone to subsequent attacks under similar circumstances, each one less likely than its predecessor to subside, and thereby increasing with their return the danger of a settled malady. I have said in all ages the phenomena of spiritual or religious excitement have been observed. We see a similarity between the proud feat of Marcus Curtius, who "loved his Rome so well," and the self-sacrificing homage

* Since this was written, I see a paper has been started in Yorkshire, as an organ for spiritual manifestations, &c.

of the worshippers of Juggernaut; we can trace in the career of the Flagellants and the victims of the dancing mania of the middle ages, points of comparison with the whirling Dervishes and the "Ranters" of our own land. If in the dark ages and among the most benighted people religious fanaticism, or phrensy, has been most frequently observed, so may we conclude, from *a priori* reasoning, it will, in like manner, be found in certain sects no less remarkable for the wild fanaticism, gross ignorance, and unscrupulous presumption of their leaders, than for the perverse enthusiasm and mental darkness of their misguided followers. The giddy, weak-minded members of such communities, led away by the impassioned, reckless teaching of their fire-brained pastors, seek to gratify their diseased religious feelings in listening to those tumultuous bursts of language, turbid streams of blasphemy and folly, which, stimulating the hearers, excite them to those disgusting and disgraceful exhibitions of maniacal fury, that may remind us indeed of the worshippers of pagan altars, who "cried aloud and cut themselves with knives and lancets," shrieking in dreadful concert, and proclaiming with hideous cries their hopes and assurance of salvation. I have been told by eye-witnesses that some of the congregation fall senseless and convulsed, others, with their eyes lit up with the fire of insanity, prophesy, leaping on benches, while the rest, in dismal wailings and prayers, signify their wants or fears. Is it at all wonderful that actual and permanent insanity should result from these wild doings? I am sure a great part of the misery and spiritual depression among the ignorant poor arises from the pernicious teaching of ranting preachers, combined with the extravagant, unmeaning, and blasphemous form of worship common to their communities. It is a significant and very instructive fact that whereas the large bulk of the inmates of public asylums may be members of the Church of England, yet it will, I believe, generally be found, at least in my experience of two public asylums it has been so, that those suffering from religious despondency, arising from religious excitement, are most frequently, if not almost invariably, members of some dissenting body—Methodists, Wesleyans, Baptists, or those, in popular phrase, termed "Ranters."

And this terminates the notice of some of the more common, and, I believe, most important causes of insanity among the poor, from the consideration of which we arrive at the inevitable conclusion, that there are certain natural laws, however unwilling man may be to receive them, beyond or against which whosoever trespasses thereby becomes subject to penalty. The fact of physical disease being thus often induced has rendered mankind, to a degree, sensible of this rule, and, in some case, to recognise the laws of health, or those general principles by which bodily suffering and sickness may be diminished, if not altogether avoided; but it seems a lamentable fact that so many fail to observe, or obey, those general rules of living that form the best safeguards to mental health and integrity. Thus, for instance, the student who sustains great intellectual labour too often succumbs to this mental strain for want of relieving his brain from severe thought, and renewing his bodily vigour by active physical exercise. Cases of intemperance, again, show the numerous instances in which mental disease results from debauchery in social life. The votary of pleasure in his carnival hour may delude himself he is the least likely ever to become insane, and yet the records of any asylum prove with how little impunity such a course of life can be indulged. To take another case; the poverty-pinched and over-wrought mechanic, whose dreary hours of toil are spent from day to day in the same small, dingy room, unpurified by the breath, unillumined by the light of heaven, and when night sets in, exhausted by confinement and want of proper food, he throws himself on his wretched pallet, and prays for sleep to soothe the pangs of famine. Could this poor man be protected from those untoward influences which threaten to destroy him, both soul and body, it is possible his dark hours would be fewer, his trials less keen; but there is no escape, and the avengers of nature's violated laws claim him at last for their victim.

When society has learned the art of preserving life and reason, we may confidently expect—though disease, whether of body or mind, will never cease to be present with us—yet its more fearful ravages shall be checked and held at bay. Even as through the Herculean exertions of humane and scientific men, those physical disorders which have in times past swept the earth like the breath of the destroying angel are now dismantled of half their terrors, and no longer permitted to go forth and destroy, so may we hope the time will come when, by the wisdom of Omniscience instructing and guiding our labours, that grim and hydra-headed monster, for which we now are content to rear costly mansions, such as few other lands can show, shall become a shadow and a name—a thing to be spoken of to our children, and to be had in memorial by our children's children in the day when they view the deserted abodes of madness and woe, and speculate on the nature and character of that terrible king for whom such palaces were designed. Well may the patriot mourn and the man of feeling sorrow to see these grisly tokens of their country's weakness rising up through the length and breadth of the land.

Let none suppose that such means, commendable though they may be for the pressing requirements of the times, will really check the spread of mental disorders. Fever wards and smallpox hospitals may shelter the sufferers of disease, but they cannot prevent it for a day; and after all, prevention, says the proverb, is better than cure; but we appoint officers of public health, whose business it is to hunt out fever and contagious maladies, the offspring of ignorance and neglect, and to trace them to their lair, and to strangle them at birth: this is far better than waging a weary fight with the full-grown monster twins, disease and death. It is wiser to go to the root and to nip the evil in the bud. And now let us think for a moment how the same principles of prevention may be applied to diseases of the mind.

Individual instances of high attainment and solitary possessions, well stored with fruitful and pleasant knowledge, will not here avail. We must have unity of action, and a combination of resources among all classes of society. For as in a well-planned arch, each single stone helps to maintain the consistence of the entire structure, so in the social constitution of our country is seen a fabric, the solid base and sturdy buttress of which are formed by the stout hearts and strong arms of the mass of labouring poor, on which firm foundation are upreared the well-polished stones, placed by the hands of the Great Architect himself, each and all in their several positions linked and bound together for mutual dependence and support, helping to sustain still higher rows of intellectual power, till the whole form an emblem of unity and strength, having Royalty for the key-stone. Until men learn the force of this truth, and recognise in obedience the principles it enjoins, we may expect to continue, as heretofore, contributing to build and fill asylums for those whose intellect has been rudely "jostled from its seat" in the angry bustle of life, unmindful how we suffer as a nation, so that as individuals we prosper,—careless if the foundations of society are sapped and destroyed, so long as we can maintain an artificial glitter in our respective places, closing, meanwhile, our eyes and ears to the miseries of those around and beneath us. Let us rather endeavour to promote mental sanitary reform, combining to introduce those changes in the social condition, more especially of the working classes, by which that high pressure system, so prejudicial to the health of the mind, shall be slackened, and the strain which it occasions relaxed. Let these people have those proper periods for repose and recreation, without which man becomes a mere machine. Let the hours of labour be abridged, and let childhood no longer share the curse of the fall. Let the multitudes who have not the means or opportunities of learning from books, be instructed by public teachers the first principles of mental as well as physical hygiene. Let them know the evils that result from ill-judged matrimonial matches, so fearfully prolific of insanity; and let not the wise examples of previous ages of the world be disregarded: the healthy manly exercises of the Olympic games, foot-races, manly contests, wrestling, throwing weights, &c., should be encouraged: the training of the body thus called forth engenders a healthy tone of mind. Would that Government prizes were instituted in every township for the successful competitors in the above-mentioned and other similar sports! Would that public ground was set apart for these healthful practices and diversions! We should not then have reason to lament for the premature decay which threatens so many of the rising generation. The tonic influence of open air would brace the nervous system, and through it the mind itself, against those ensnaring and degrading vices into which the youth of our nation fall. A manly spirit of emulation would arise to preserve their stamina unspoiled, their physical resources unalloyed, and hence not to themselves only, but to their posterity would accrue the advantages of a well-trained body, with the blessing of a strong and healthy mind. That such a day may dawn, when England's sons shall learn the prowess and strength of nerve for which their forefathers were famed,—when, forsaking the tavern and gambling-table, the low haunts of vice and idleness, they shall acquire a taste for those spirited and adventurous pursuits by which not men but heroes are formed, let each one who has the power feel the responsibility, and seek without delay to bring about so desirable a consummation.

---

